# Associations of lifestyle with mental health and well-being in Chinese adults: a nationwide study

**DOI:** 10.3389/fnut.2023.1198796

**Published:** 2023-06-23

**Authors:** Xue Wang, Yibo Wu, Xinji Shi, Yu Chen, Yahong Xu, Hongbo Xu, Yanan Ma, Shuang Zang

**Affiliations:** ^1^Department of Community Nursing, School of Nursing, China Medical University, Shenyang, Liaoning, China; ^2^School of Public Health, Peking University, Beijing, China; ^3^School of Nursing, Southern Medical University, Guangzhou, Guangdong, China; ^4^Department of Fundamental of Nursing, School of Nursing, Capital Medical University, Beijing, China; ^5^Department of Medical Nursing, School of Nursing, Wenzhou Medical University, Wenzhou, Zhejiang, China; ^6^Department of Biostatistics and Epidemiology, School of Public Health, China Medical University, Shenyang, Liaoning, China

**Keywords:** mental health, adults, China, lifestyle, well-being

## Abstract

**Background:**

A healthy lifestyle is beneficial to individuals’ health. However, little is known about the associations of lifestyle factors with mental health and well-being. This study examined the associations of lifestyle factors with mental health (i.e., depression, anxiety, loneliness, perceived pressure, and self-rated health status) and well-being in Chinese adults.

**Methods:**

A nationally representative survey was conducted in China from 20 June 2022 to 31 August 2022. Data from the survey were analyzed using multiple linear regression to determine the associations of lifestyle with mental health and well-being in Chinese adults. Standardized regression coefficients (β) and 95% confidence intervals (CIs) were estimated using multiple linear regression.

**Results:**

The survey included 28,138 Chinese adults. Multiple linear regression results showed that there were significant negative associations of lifestyle scores with scores of depression (*β* = −0.93, 95% CI: −0.98, −0.88), anxiety (*β* = −0.71, 95% CI: −0.76, −0.67), loneliness (*β* = −0.23, 95% CI: −0.24, −0.21), and perceived pressure (*β* = −0.19, 95% CI: −0.22, −0.16). Moreover, there were significant positive associations of lifestyle with self-rated health status (*β* = 1.99, 95% CI: 1.79, 2.20) and well-being (*β* = 0.96, 95% CI: 0.91, 1.02).

**Conclusion:**

This study provides insight into the associations of lifestyle factors with mental health and well-being and highlights the importance of improving and maintaining healthy lifestyle behaviors for favorable mental health and well-being.

## Introduction

Mental health is the foundation for overall health and well-being (1). According to the World Health Organization (WHO), mental health problems are prevalent worldwide, affecting approximately one in four individuals at some point during their lifetime (2). Globally, approximately one-third of adult populations experience mental health problems annually, and these problems are a significant public health challenge (3, 4). As such, addressing mental health issues has become a vital priority for public health programs, including treatment, prevention, and health promotion efforts (5). Given the significant burden of mental health problems on individuals and society as a whole, raising public awareness of the importance of mental health is crucial.

### Literature review

Previous research has shown that some daily behaviors could be adapted to affect mental health (6, 7). Mental health programs that encourage individuals to manage their mental health by adjusting daily health behaviors are destigmatizing, empowering, and cost-effective in affecting the population (8). Several nationwide studies have identified some lifestyle factors associated with mental health, including physical activity, healthy eating behaviors, and adequate sleep, which are associated with reduced depression, anxiety, and stress levels (9–11). Conversely, some nationwide studies also found that increased consumption of smoking and alcohol is associated with unfavorable mental health outcomes (12–14). However, research on the association between lifestyle factors and positive psychological outcomes such as well-being is limited, despite some nationwide studies indicating that setting time, smoking, and diet are individually associated with well-being among adults (15–17). It is important to note that previous nationwide studies have mostly focused on solely behaviors, with limited research on the combination of these lifestyle factors. While analyzing these factors independently provides valuable information, combining them may better reflect real-life situations, as they often co-occur and may have synergistic effects (18, 19). Additionally, since lifestyle factors are multidimensional and complex, a comprehensive analysis of these factors may be more effective in capturing their association with mental health and well-being, compared to focusing on solely factors (20).

### Theoretical underpinning

The present study is theoretically anchored in the biopsychosocial model of health and the health promotion model. These theoretical frameworks provide a comprehensive and holistic understanding of the intricate associations between lifestyle factors and mental health and well-being. The biopsychosocial model of health underscores the dynamic interplay among biological, psychological, and social factors in determining an individual’s health status (21). In the context of this study, this model elucidates the potential correlation of lifestyle factors, such as diet and exercise, with mental health and well-being. The health promotion model accentuates the elements that facilitate and maintain healthy behaviors, highlighting the importance of individual perceptions, motivations, and behaviors in shaping health outcomes (22). This model offers insights into the psychological and behavioral mechanisms underlying the associations between lifestyle factors and mental health and well-being. Together, these two theoretical frameworks furnish a robust foundation for exploring the intricate associations between lifestyle and mental health and well-being in the general population and have significant implications for the development of effective interventions and policies to promote mental health and well-being in this population.

Thus, the aim of this nationally representative survey was to explore whether seven lifestyle factors (i.e., smoking, drinking, diet behaviors, physical activity, sitting time, sleep duration, and sleep quality) were associated with mental health (i.e., depression, anxiety, loneliness, perceived pressure, and self-rated health status) and well-being. This study aims to fill the research gap related to a nationwide survey and contribute to the development of effective health promotion programs and policies to improve mental health and well-being.

## Methods

### Survey design and study population

This survey was conducted by a multistage sampling method across 31 (91.17% of the total) provinces/autonomous regions/municipalities/special administrative regions in China from 20 June 2022 to 31 August 2022. In this survey, investigators issued one-on-one questionnaires to participants by using the online Questionnaire Star platform. The inclusion criteria for the study participants were as follows: Chinese people ≥18 years old who participated in the study voluntarily, understood the meaning of each questionnaire item, and completed the questionnaires independently. For participants with thinking ability but without sufficient mobility to complete the questionnaire, investigators assisted without intervening. The exclusion criteria for the study participants were as follows: people with confusion and mental disorders, participation in other similar studies, and refusal to participate. The participants who were diagnosed with mental illness by a doctor were identified through a combination of self-report and systematic record-keeping (available in community health service centers). During the survey, community workers (neighborhood committees or health service center staff) who were familiar with the local community also participated. Based on these criteria, respondents with psychological problems were excluded from the data pool. The specific survey process and quality control information were described in a previous study (23).

This study was approved by the Ethics Research Committee of the Health Culture Research Center of Shaanxi (No. JKWH-2022-02), and all participants provided informed consent before data collection.

### Exposure measure

#### Lifestyle score

According to the WHO report and prior studies (24–26), we selected seven lifestyle factors to construct a lifestyle score. These lifestyle factors included smoking, drinking, diet behaviors, physical activity, sitting time, sleep duration, and sleep quality.

First, the smoking status of participants was measured by a single item classified into a lower-risk category (former and never smoking) or a higher-risk category (current smoking) (27). Second, the drinking status of participants was assessed by a single item classifying them into a lower-risk category (former and never drinking) or a higher-risk category (current drinking) (28). Third, diet behaviors were determined by examining breakfast behavior, tea-drinking behavior, sugar-sweetened beverage drinking behavior, eating out behavior, and water drinking behavior (29–31). For example, if the participants ate breakfast daily or 5–6 days/week, they were classified into a lower-risk category; otherwise, they were classified into a higher-risk category. Detailed information and coding methods on diet behavior ratings are presented in Supplementary Table S1. Then, a diet score was generated according to the above diet behaviors, with a score ≥3 indicating low-risk diet behavior. Fourth, the participants’ physical activity levels were evaluated by the International Physical Activity Questionnaire-7 (IPAQ-7) (32), and we then calculated individuals’ basal metabolic time per week (minute). The following calculation method was used: (1) Mild-intensity activity metabolic equivalent of task (MET) = 3.3 × average time engaged in mild-intensity activity daily × weekly engaging in mild-intensity activity days. (2) Moderate-intensity activity MET = 4.0 × average time engaged in moderate-intensity activity daily × weekly engaging in moderate-intensity activity days. (3) Strenuous activity MET = 8.0 × average time engaged in strenuous activity daily × weekly engaging in strenuous activity days. Thus, basal metabolic time weekly (minute) = (1) + (2) + (3). Then, the physical activity of participants was classified into a lower-risk category [active (≥3,000 MET)] or a higher-risk category [minimally active (≥600 and <3,000 MET) and inactive (<600 MET)] (33). Fifth, the sitting time of participants was assessed by a single item, and the participants were classified into a lower-risk category (≤7 h/day) or a higher-risk category (>7 h/day) (34). Sixth, the sleep duration of participants was classified into lower-risk (>7 h/day) or higher-risk categories (≤7 h/day) (35). Finally, the sleep quality of participants was classified into lower-risk (relatively good and very good) or higher-risk categories (relatively bad and very bad) (36). The specific coding for these lifestyle factors is shown in Supplementary Table S2.

The above seven lifestyle factors were combined into a lifestyle score (ranging from 0 to 7), with higher scores representing healthier lifestyles. Due to the distribution of data, lifestyle was further categorized into five groups by the score (0–2, 3, 4, 5, and 6–7).

### Outcome measure

#### Depression

The Patient Health Questionnaire-9 (PHQ-9) is used to measure participants’ depression status (37). Each item is scored on a four-point Likert scale, ranging from 0 (never) to 3 (nearly every day). The total score of the PHQ-9 ranges from 0 to 27, with higher scores representing more severe depression. The Cronbach’s α for the PHQ-9 was 0.920 in this study.

#### Anxiety

The Generalized Anxiety Disorder-7 (GAD-7) is used to assess participants’ anxiety status (38). Each item is scored on a four-point Likert scale, ranging from 0 (never) to 3 (nearly every day). The total score of the GAD-7 ranges from 0 to 21, with higher scores reflecting more severe anxiety. The Cronbach’s α for the GAD-7 was 0.944 in this study.

#### Loneliness

The Three-Item Loneliness Scale (T-ILS) is used to evaluate participants’ loneliness (39). Each item is scored on a three-point Likert scale, ranging from 1 (never) to 3 (often). The total score of the T-ILS ranges from 3 to 9, with higher scores indicating higher levels of loneliness. The Cronbach’s α for the T-ILS was 0.861 in this study.

#### Perceived pressure

The Perceived Stress Scale-4 (PSS-4) is used to measure participants’ perceived pressure (40). Each item is scored on a five-point Likert scale, ranging from 1 (never) to 5 (always). The total score of the PSS-4 ranges from 4 to 20, with higher scores representing greater perceived pressure. The Cronbach’s α for the PSS-4 was 0.681 in this study.

#### Self-rated health status

Participants rated their health status on a vertical scale of 0 (the least healthy) to 100 (the healthiest) (41).

#### Well-being

The World Health Organization Well-Being Index-5 (WHO-5) is used to evaluate participants’ psychological well-being (42). Each item is scored on a six-point Likert scale, ranging from 0 (never before) to 5 (all times). The total score of the WHO-5 ranges from 0 to 25, with higher scores reflecting greater well-being. The Cronbach’s α for the WHO-5 was 0.951 in this study.

### Covariates

The following variables were included as covariates: age, sex, education level (junior high school and below, high school and junior college, bachelor’s degree and above), career status (student, have a job, have no job), marital status (have no partner, have a partner), urban–rural distribution, whether having diagnosed chronic disease, family *per capita* monthly income (≤3,000 Chinese Yuan (CNY), 3001–6000 CNY, ≥6001 CNY), and self-rated family social status (scoring from 1 (lowest) to 7 (highest)).

### Statistical analysis

First, Kolmogorov–Smirnov tests were performed to determine the normality of continuous variables. The continuous variables in this study were approximately normally distributed according to visual inspection of Q-Q plots. Second, the distributions of participant characteristics were examined based on the categories of lifestyle scores. Continuous variables were displayed as the mean and standard deviation (SD), and categorical variables were presented as numbers and percentages. The chi-squared test was performed to compare the categorical variables among lifestyle score groups, and variance analysis was conducted to compare the continuous variables. The collinearity between variables was determined by measuring the variance inflation factor (VIF). The multicollinearity test demonstrated no collinearity among the study variables in this study (maximum VIF = 2.53). Third, the associations of lifestyle with mental health and well-being were conducted using multiple linear regression adjusting for all covariates. Fourth, we generated fitting plots using generalized additive models to depict the associations of lifestyle with mental health and well-being, with adjustment for potential confounders. Fifth, the distribution of mental health and well-being scores in the lifestyle score groups was visualized using violin plots. Sixth, we also used violin plots to visualize the distribution of mental health and well-being scores in the lifestyle score groups stratified by categorical variables of covariates. Finally, the associations of lifestyle factors and detailed lifestyle behaviors with mental health and well-being were conducted by using multiple linear regression with all covariates adjusted.

All statistical tests were two-sided, and the significance level was set at *p* < 0.05. All statistical analyses were performed using Stata version 16.0 (StataCorp, College Station, TX, United States).

## Results

### Participant characteristics

A total of 28,138 Chinese adults were included in this survey. In this study, 43.00% of participants were male, 41.83% had a bachelor’s degree and above, 49.80% had no partner, and 72.43% lived in urban areas. There were significant differences in demographic characteristics among the different lifestyle score groups (all *p* < 0.05) (Table 1). The mean score of the depression scale among the participants was 6.87 (SD: 5.61) points, the anxiety scale was 4.99 (SD: 4.72) points, the loneliness scale was 4.67 (SD: 1.63) points, the perceived pressure scale was 10.10 (SD: 3.09) points, the self-rated health status scale was 72.65 (SD: 22.40) points, and the well-being scale was 14.25 (SD: 6.09) points. Out of the 28,138 participants, 13.32% (*n* = 3,749) had a higher-risk smoking status, 21.81% (*n* = 6,138) had a higher-risk drinking status, 41.91% (*n* = 11,793) had higher-risk diet behaviors, 52.22% (*n* = 14,695) had higher-risk physical activity, and 47.36% (*n* = 13,325) had higher-risk sitting time. Additionally, 65.34% (*n* = 18,385) had higher-risk sleep duration, and 17.50% (*n* = 4,924) had higher-risk sleep quality (Table 2). Among the 28,138 participants, 2023 participants (7.19%) had a lifestyle score of 0–2, 4,436 participants (15.77%) had a lifestyle score of 3, 7,866 participants (27.96%) had a lifestyle score of 4, 8,231 participants (29.25%) had a lifestyle score of 5, and 5,582 participants (19.84%) had a lifestyle score of 6–7 (Table 2) (Supplementary Figure S1). Participants with lower lifestyle scores were more likely to have higher scores for depression, anxiety, loneliness, and perceived pressure. Participants with higher lifestyle scores tended to have higher scores for self-rated health status and well-being (Supplementary Figure S2). The distribution of mental health and well-being scores by lifestyle score stratified by categorical variables of covariates can be seen in Supplementary Figures S3–S9.

**Table 1:** Characteristics of participants according to lifestyle score (*n* = 28,138).

Variables	Total sample	Lifestyle score					
		0–2	3	4	5	6–7	*p*
Age, mean (SD)	37.25 (17.83)	32.88 (15.80)	34.32 (16.81)	35.18 (17.36)	38.76 (18.19)	41.83 (18.18)	<0.001
Sex, *n* (%)							<0.001
Male	12,099 (43.00)	1,180 (58.33)	2,209 (49.80)	3,347 (42.55)	3,187 (38.72)	2,176 (38.98)	
Female	16,039 (57.00)	843 (41.67)	2,227 (50.20)	4,519 (57.45)	5,044 (61.28)	3,406 (61.02)	
Education level, *n* (%)							<0.001
Junior high school and below	6,334 (22.51)	305 (15.08)	792 (17.85)	1,498 (19.04)	2014 (24.47)	1725 (30.90)	
High school and junior college	10,034 (35.66)	787 (38.90)	1,579 (35.60)	2,786 (35.42)	2,943 (35.76)	1939 (34.74)	
Bachelor degree and above	11,770 (41.83)	931 (46.02)	2065 (46.55)	3,582 (45.54)	3,274 (39.78)	1918 (34.36)	
Career status, *n* (%)							<0.001
Student	10,023 (35.62)	816 (40.34)	1837 (41.41)	3,236 (41.14)	2,687 (32.64)	1,447 (25.92)	
Have no job	5,656 (20.10)	262 (12.95)	664 (14.97)	1,371 (17.43)	1908 (23.18)	1,451 (25.99)	
Have a job	12,459 (44.28)	945 (46.71)	1935 (43.62)	3,259 (41.43)	3,636 (44.17)	2,684 (48.08)	
Marital status, *n* (%)							<0.001
Have no partner	14,013 (49.80)	1,254 (61.99)	2,581 (58.18)	4,308 (54.77)	3,785 (45.98)	2085 (37.35)	
Have a partner	14,125 (50.20)	769 (38.01)	1855 (41.82)	3,558 (45.23)	4,446 (54.02)	3,497 (62.65)	
Urban–rural distribution, *n* (%)							<0.001
Rural	7,757 (27.57)	468 (23.13)	1,150 (25.92)	2060 (26.19)	2,357 (28.64)	1722 (30.85)	
Urban	20,381 (72.43)	1,555 (76.87)	3,286 (74.08)	5,806 (73.81)	5,874 (71.36)	3,860 (69.15)	
Whether having diagnosed chronic disease, *n* (%)							<0.001
No	21,501 (76.41)	1,450 (71.68)	3,318 (74.80)	6,062 (77.07)	6,308 (76.64)	4,363 (78.16)	
Yes	6,637 (23.59)	573 (28.32)	1,118 (25.20)	1804 (22.93)	1923 (23.36)	1,219 (21.84)	
Family *per capita* monthly income (Chinese Yuan), *n* (%)							<0.001
≤3,000	9,503 (33.77)	602 (29.76)	1,573 (35.46)	2,659 (33.80)	2,769 (33.64)	1900 (34.04)	
3,001–6,000	11,334 (40.28)	804 (39.74)	1720 (38.77)	3,115 (39.60)	3,397 (41.27)	2,298 (41.17)	
≥6,001	7,301 (25.95)	617 (30.50)	1,143 (25.77)	2092 (26.60)	2065 (25.09)	1,384 (24.79)	
Family social status (scores), mean (SD)	4.31 (1.31)	4.00 (1.36)	4.17 (1.31)	4.29 (1.32)	4.40 (1.28)	4.42 (1.27)	<0.001

Total percentages within categories may not equal 100% due to rounding.

**Table 2:** Characteristics of mental health, well-being, and lifestyle factors of the participants (*n* = 28,138).

Variables	Value
**Mental health and well-being**	
Depression scale (scores), mean (SD)	6.87 (5.61)
Anxiety scale (scores), mean (SD)	4.99 (4.72)
Loneliness scale (scores), mean (SD)	4.67 (1.63)
Perceived pressure scale (scores), mean (SD)	10.10 (3.09)
Self-rated health status scale (scores), mean (SD)	72.65 (22.40)
Well-being scale (scores), mean (SD)	14.25 (6.09)
**Lifestyle factors**	
**Smoking status, *n* (%)**	
Higher-risk	3,749 (13.32)
Lower-risk	24,389 (86.68)
**Drinking status, *n* (%)**	
Higher-risk	6,138 (21.81)
Lower-risk	22,000 (78.19)
**Diet behaviors, *n* (%)**	
Higher-risk	11,793 (41.91)
Lower-risk	16,345 (58.09)
**Physical activity, *n* (%)**	
Higher-risk	14,695 (52.22)
Lower-risk	13,443 (47.78)
**Sitting time, *n* (%)**	
Higher-risk	13,325 (47.36)
Lower-risk	14,813 (52.64)
**Sleep duration, *n* (%)**	
Higher-risk	18,385 (65.34)
Lower-risk	9,753 (34.66)
**Sleep quality, *n* (%)**	
Higher-risk	4,924 (17.50)
Lower-risk	23,214 (82.50)
**Lifestyle score**	
0–2, *n* (%)	2,023 (7.19)
3, *n* (%)	4,436 (15.77)
4, *n* (%)	7,866 (27.96)
5, *n* (%)	8,231 (29.25)
6–7, *n* (%)	5,582 (19.84)

Total percentages within categories may not equal 100% due to rounding.

### Associations of lifestyle with mental health and well-being

**Figure 1:**
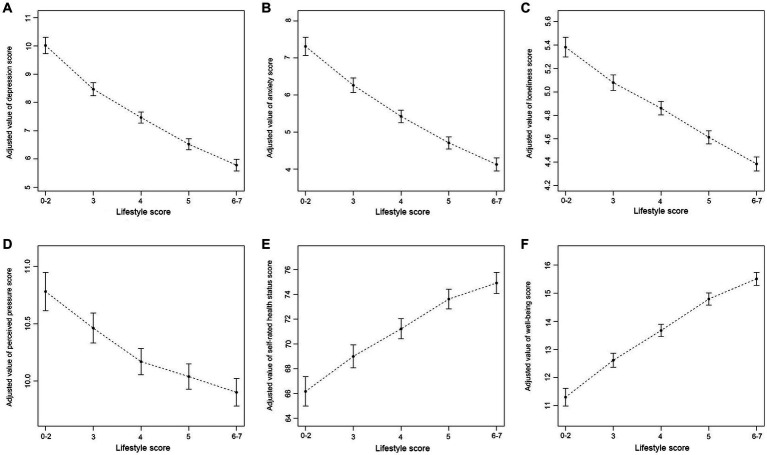
Associations of lifestyle with mental health and well-being. **(A)** Association of lifestyle with depression; **(B)** Association of lifestyle with anxiety; **(C)** Association of lifestyle with loneliness; **(D)** Association of lifestyle with perceived pressure; **(E)** Association of lifestyle with self-rated health status; **(F)** Association of lifestyle with well-being. All models were adjusted for age, sex, education level, career status, marital status, urban–rural distribution, whether having diagnosed chronic disease, family *per capita* monthly income, and family social status.

The multiple linear regression results showed that there were significant negative associations between lifestyle score (as a continuous variable) and scores of depression (*β* = −0.93, 95% CI: −0.98, −0.88), anxiety (*β* = −0.71, 95% CI: −0.76, −0.67), loneliness (*β* = −0.23, 95% CI: −0.24, −0.21), and perceived pressure (*β* = −0.19, 95% CI: −0.22, −0.16). Moreover, there were significant positive associations of lifestyle score (as a continuous variable) with scores of self-rated health status (*β* = 1.99, 95% CI: 1.79, 2.20) and well-being (*β* = 0.96, 95% CI: 0.91, 1.02). When the lifestyle score was treated as a categorical variable, all results were statistically significant (Table 3). After adjusting for possible confounders, negative associations between lifestyle score (as a categorical variable) and scores of depression, anxiety, loneliness, and perceived pressure were observed. Similarly, lifestyle scores were positively associated with scores of self-rated health status and well-being (see Figure 1 for details).

**Table 3:** Associations of lifestyle with mental health and well-being (n = 28,138).

Items	Mental health										Well-being	
	Depression		Anxiety		Loneliness		Perceived pressure		Self-rated health status			
	*β* (95%CI)	*P*	*β* (95%CI)	*P*	*β* (95%CI)	*P*	*β* (95%CI)	*P*	*β* (95%CI)	*P*	*β* (95%CI)	*P*
**Continuous**												
Lifestyle score	−0.93 (−0.98, −0.88)	<0.001	−0.71 (−0.76, −0.67)	<0.001	−0.23 (−0.24, −0.21)	<0.001	−0.19 (−0.22, −0.16)	<0.001	1.99 (1.79, 2.20)	<0.001	0.96 (0.91, 1.02)	<0.001
**Categorical**												
**Lifestyle score**												
0–2	1 (ref)		1 (ref)		1 (ref)		1 (ref)		1 (ref)		1 (ref)	
3	−1.56 (−1.84, −1.28)	<0.001	−1.06 (−1.30, −0.82)	<0.001	−0.31 (−0.39, −0.23)	<0.001	−0.32 (−0.48, −0.16)	<0.001	2.85 (1.70, 4.01)	<0.001	1.32 (1.01, 1.63)	<0.001
4	−2.56 (−2.83, −2.30)	<0.001	−1.90 (−2.13, −1.68)	<0.001	−0.53 (−0.60, −0.45)	<0.001	−0.61 (−0.76, −0.46)	<0.001	5.07 (4.00, 6.15)	<0.001	2.38 (2.09, 2.67)	<0.001
5	−3.51 (−3.77, −3.24)	<0.001	−2.61 (−2.84, −2.39)	<0.001	−0.77 (−0.85, −0.70)	<0.001	−0.74 (−0.89, −0.59)	<0.001	7.47 (6.39, 8.55)	<0.001	3.50 (3.21, 3.79)	<0.001
6–7	−4.26 (−4.53, −3.98)	<0.001	−3.21 (−3.44, −2.97)	<0.001	−1.00 (−1.08, −0.92)	<0.001	−0.88 (−1.04, −0.72)	<0.001	8.78 (7.65, 9.92)	<0.001	4.22 (3.91, 4.52)	<0.001

All models were adjusted for age, sex, education level, career status, marital status, urban–rural distribution, whether having diagnosed chronic disease, family per capita monthly income, and family social status.

β, regression coefficients; CI, confidence interval; ref, reference.

Additionally, after adjusting for potential confounders, the results of multiple linear regression indicated that significant associations between lifestyle factors and most of the outcomes remained robust. For example, when examining the associations of smoking status with scores of depression, anxiety, loneliness, perceived pressure, self-rated health status, and well-being, the β value (vs. higher-risk) was −1.19, −0.85, −0.18, −0.17, 2.16, and 1.31 for lower-risk, respectively. Similarly, lower-risk sitting time was associated with lower scores for depression (*β* = −1.13), anxiety (*β* = −0.92), and loneliness (*β* = −0.28) but with higher scores for self-rated health status (*β* = 1.75) and well-being (*β* = 0.25) than higher-risk sitting time. In addition, when analyzing the associations of sleep duration with scores of depression, anxiety, loneliness, perceived pressure, self-rated health status, and well-being, the β value (vs. higher-risk) was −1.24, −1.00, −0.34, −0.22, 3.24, and 1.55 for lower-risk, respectively. Lower-risk sleep quality was associated with lower scores of depression (*β* = −3.69), anxiety (*β* = −2.93), and loneliness (*β* = −0.79) but with higher scores of self-rated health status (*β* = 8.96) and well-being (*β* = 3.15) compared to the higher-risk sleep quality group (see Supplementary Table S3). Similarly, when analyzing the associations of detailed lifestyle behaviors with mental health and well-being, most results (e.g., diet scores and sitting time) were statistically significant (see Supplementary Table S4).

## Discussion

The current study explored the associations of lifestyle with mental health and well-being. The results showed significant negative associations of lifestyle scores with scores of depression, anxiety, loneliness, and perceived pressure. Moreover, there were significant positive associations of lifestyle scores with scores of self-rated health status and well-being. In this field, Firth et al. (43) conducted a meta-analysis and found that lifestyle factors such as exercise, smoking, diet, and sleep were closely associated with mental health. This study was conducted with the primary objective of investigating the association between lifestyle and mental health and well-being, while considering multiple outcomes, including depression, anxiety, loneliness, and perceived pressure. Our study, to some extent, served to complement outcomes (e.g., loneliness and self-rated health status) that were not comprehensively addressed in Firth’s meta-analysis. In addition, our study revealed positive associations between lifestyle scores and both self-rated health status and well-being. These novel findings provide deeper insights into the complex associations between lifestyle and mental health and well-being, enriching the understanding of this topic. Our study emphasizes the critical importance of combining lifestyle factors with mental health and well-being research and offers new evidence to support the existing studies.

The depression and anxiety scale scores in our study were slightly lower than the scores reported in previous nationwide population-based studies conducted in Turkey, Germany, and Poland (44–46). Regarding subjective well-being, the scores of our study were higher than those obtained in previous studies conducted in 15 European countries (47). The observed differences may be attributed to cultural variations, including the importance placed on interpersonal harmony and emotional restraint in China (48, 49). Additionally, variations in study design and sampling methods may also have played a role. As well, differences in socioeconomic context and healthcare systems could also impact mental health outcomes. Further investigation is needed to better understand the underlying factors contributing to these disparities. However, it is worth noting that variations in theoretical foundations and research themes may result in different studies incorporating diverse lifestyle indicators (50, 51), which can make cross-study comparisons challenging.

In this study, we found that participants with lower lifestyle scores tended to have higher scores of depression and anxiety, which was similar to other studies (50, 52). The evidence suggests that individuals’ lifestyle factors, such as smoking status (53), diet behaviors (54), physical activity (55), sedentary behavior (56), and alcohol consumption (57), are associated with depression and anxiety status. Lifestyle factors may be associated with depression and anxiety via multiple pathways, including modifying neurotrophins essential to psychological disorders as well as nitrosative and oxidative stress pathways (58, 59). Additionally, individuals with depression and anxiety tend to have higher systemic inflammation levels (60). Higher systemic inflammation levels have also been demonstrated to be associated with unfavorable lifestyle factors, including unhealthy diet behaviors (61), low physical activity levels (62), and smoking (63). The present findings provide support for the growing evidence linking lifestyle factors to mental health outcomes and emphasize the critical role of lifestyle in the prevention of depression and anxiety. Encouraging individuals to adopt a healthier lifestyle, including smoking cessation, healthy dietary habits, regular physical activity, and limiting sedentary behavior and alcohol consumption, could help reduce the risk of depression and anxiety and improve overall mental health. Healthcare providers should also prioritize assessing and addressing lifestyle factors as part of their management approach to depression and anxiety.

The results of our study revealed that lifestyle was negatively associated with perceived pressure, which was in accordance with prior studies (64, 65). Individuals were inclined to engage in less tiresome activities during stressful times and avoided physical activity, probably due to time constraints and limited self-regulation capabilities (66, 67). Additionally, studies have also suggested that individuals often practice unhealthy behaviors to cope with emotion-focused stress, including smoking, drinking, reducing sleep duration, or avoiding physical activity (65, 68). Stress appears to be associated with eating behavior changes in an unhealthy direction (69). Moreover, work and academic stress are pervasive among adults (70). Therefore, public health policies ought to advocate for individuals to maintain or enhance healthy lifestyle practices to obtain maximum benefit from potential stress buffering and stress management. This goal can be realized through educational programs and interventions that aim to reduce unhealthy behaviors while promoting healthy habits.

Our study discovered that individuals with lower lifestyle scores tend to have higher loneliness levels. Research has reported that a sedentary lifestyle might increase the risk of loneliness (71). Several studies have also suggested that loneliness is associated with adverse health behaviors (e.g., less physical activity), poorer health practices (e.g., smoking and alcohol consumption) (72, 73), and sleep disturbances (e.g., decreased sleep duration and poorer sleep quality) (74, 75). In addition, as an important relevant factor for health, self-regulation ability may be one explanatory factor for poorer health behaviors in lonely individuals. A previous study showed that poorer self-regulation ability was associated with adults’ loneliness (76). Poor self-regulation ability may contribute to loneliness-related health risks via reduced participation in health-promoting behaviors (73). Furthermore, poorer self-regulation ability often accompanies unhealthy lifestyles in adults (77). The results of our study highlight the importance of promoting individual responsibility for health among lonely populations, including participation in healthy lifestyle behaviors. These findings suggest that public health policies should focus on promoting healthy lifestyle practices to reduce loneliness levels and associated negative health behaviors. Targeted interventions are especially crucial for individuals with poor self-regulation abilities. Moreover, those in need of support should seek out resources such as social support and psychological counseling to decrease loneliness levels and improve overall health.

The study showed that lifestyle was positively associated with self-rated health status, consistent with previous findings (78, 79). Self-rated health status is a multidimensional concept. For individuals, it was associated with a multifactorial composite representing personal, psychological, social, medical, and behavioral characteristics (80, 81). Similarly, sedentary behaviors, sleep duration, diet behaviors, physical activity, alcohol, and smoking consumption were associated with individuals’ health outcomes (82, 83). Thus, promoting healthy behaviors holistically rather than separately is an effective public health strategy for improving health, in general, and self-rated health status, in particular. In practice, this means that public health interventions should consider multiple dimensions of an individual’s health and well-being. For example, interventions addressing both physical activity and social isolation, rather than treating them separately, could be more effective in promoting healthy behavior. Such a comprehensive approach may help public health practitioners develop more effective strategies for improving health outcomes and self-rated health status.

Moreover, our study revealed that lifestyle was positively associated with higher levels of well-being. Previous studies also found similar trends (7, 29). Individuals with high physical activity levels (84), lower-risk sitting time (85), and healthier dietary behaviors (17) were more likely to have increased well-being. The findings of this study underscored that more attention should be given to associations between lifestyle behaviors and individual well-being. This study provides valuable insights for healthcare professionals that adopting healthier lifestyles, including increased physical activity levels, reduced prolonged sedentary time, and healthier dietary habits, represents a meaningful avenue for enhancing individual well-being. By incorporating these behaviors into public health interventions, healthcare professionals can effectively promote positive health outcomes and improve overall well-being. Furthermore, by recognizing the associations between lifestyle behaviors and individual well-being, healthcare professionals can develop more comprehensive and holistic strategies to address the multifaceted nature of health and well-being.

## Conclusion

In this nationwide study, we found negative associations of lifestyle scores with scores of depression, anxiety, loneliness, and perceived pressure and positive associations of lifestyle scores with scores of self-rated health status and well-being. These findings suggest that the adoption of a multi-behavioral healthy lifestyle, rather than just focusing on single behaviors, may be an effective approach to promoting and maintaining mental health and well-being.

The limitations of this study should also be acknowledged. First, it is essential to note that, similar to other studies, due to limitations of the cross-sectional design, causality cannot be identified. Thus, mental health and well-being could be the results or causes of a lifestyle. There is a necessity for further longitudinal and prospective studies to determine these associations. Second, all information was self-reported, meaning that it may not always reflect real situations. Some variables, such as smoking behavior, may tend to be underestimated. Third, while the findings from this study may be applicable to other countries’ health promotion programs, it is still imperative that these findings are tested in other social contexts since the current findings were solely restricted to studying the Chinese adult sample. Finally, in our study, we analyzed PHQ and GAD scores as continuous variables, following the methods employed in previous studies (86, 87). However, it is crucial to exercise caution when using these scores as continuous variables, particularly with scores that fall below the established cutoff points. Such scores may not always indicate the absence of depression and anxiety, and changes in scores cannot be simply interpreted as better or worse symptoms. Although using PHQ and GAD scores as continuous variables is a commonly utilized method, other measurement tools and methods may be explored in future research to enhance the accuracy and effectiveness of evaluating depression and anxiety symptoms.

Despite these limitations, our findings have important implications for the field of public health. These findings highlight the need to integrate the promotion of a healthy lifestyle into mental health promotion programs. Our results suggest that policymakers and healthcare professionals should take a comprehensive approach to promoting a healthy lifestyle and its positive effects on mental health and well-being. This includes encouraging individuals to engage in multiple healthy behaviors, such as physical activity, a healthy diet, and reducing sedentary behavior.

Our study contributes to the broader literature by providing evidence for the importance of a multi-behavioral healthy lifestyle in promoting mental health and well-being. The findings support and extend previous research in this field and underscore the need for further research on the association between lifestyle behaviors and mental health outcomes.

Overall, our study emphasizes the significance of promoting a healthy lifestyle and its potential to improve mental health and well-being. It underscores the need for healthcare professionals and policymakers to develop effective strategies to promote healthy behaviors, particularly in the context of mental health promotion programs.

## Data availability statement

The original contributions presented in the study are included in the article/Supplementary material, further inquiries can be directed to the corresponding author.

## Ethics statement

This study was approved by the Ethics Research Committee of the Health Culture Research Center of Shaanxi (No. JKWH-2022-02). The patients/participants provided their written informed consent to participate in this study.

## Author contributions

XW: conceptualization, methodology, formal analysis, writing–original draft, and reviewing and editing. YW: data collection, methodology, writing–original draft, reviewing and editing. XS, YC, YX, HX, and YM: and reviewing and editing. SZ: conceptualization and reviewing and editing. All authors contributed to the article and approved the submitted version.

## Funding

This study was supported by the 2020 Scientific Research Funding Project of Education Department of Liaoning Province (No. QNRW2020003).

## Conflict of interest

The authors declare that the research was conducted in the absence of any commercial or financial relationships that could be construed as a potential conflict of interest.

## Publisher’s note

All claims expressed in this article are solely those of the authors and do not necessarily represent those of their affiliated organizations, or those of the publisher, the editors and the reviewers. Any product that may be evaluated in this article, or claim that may be made by its manufacturer, is not guaranteed or endorsed by the publisher.
