# Supplementary material for: Associations of lifestyle with mental health and well-being in Chinese adults: a nationwide study
Source: Front Nutr. 2023 Jun 23;10:1198796. doi: 10.3389/fnut.2023.1198796 (PMC10327438; doi:10.3389/fnut.2023.1198796)
Supplement: Supplementary file 1 [file Data_Sheet_1.PDF]

Supplementary Table 1 online only. Components and scoring classification for diet behaviors

Diet behaviors	Classification	Score
Breakfast behavior	Daily	1
	5-6 days/week	1
	3-4 days/week	0
	1-2 days/week	0
	Never	0
Tea-drinking behavior	Current	1
	Never	0
Sugar-sweetened beverage drinking behavior	Never	1
	1-3 bottles/week	0
	≥4 bottles/week	0
Eating out behavior	Never	1
	1-2 times/week	0
	≥3 times/week	0
Water drinking behavior	≥1200 ml/day	1
	<1200 ml/day	0

Note: 1 bottle=300 ml.

Supplementary Table 2 online only. Combined lifestyle score in Chinese adults

Lifestyle factors	Risk classification	Detailed behaviors classification	Score
Smoking status	Higher-risk	Current	0
	Lower-risk	Former	1
		Never	1
Drinking status	Higher-risk	Current	0
	Lower-risk	Former	1

		Never	1
Diet behaviors	Higher-risk	Diet scores < 3	0
	Lower-risk	Diet scores ≥ 3	1
Physical activity	Higher-risk	Inactive (< 600 MET)	0
		Minimally active (≥ 600 and < 3000 MET)	0
	Lower-risk	Active (≥ 3000 MET)	1
Sitting time	Higher-risk	> 7 hours/day	0
	Lower-risk	≤ 7 hours/day	1
Sleep duration	Higher-risk	< 5 hours/day	0
		5-6 hours/day	0
		6-7 hours/day	0
	Lower-risk	>7 hours/day	1
Sleep quality	Higher-risk	Very bad	0
		Relatively bad	0
	Lower-risk	Relatively good	1
		Very good	1

MET, metabolic equivalent of task.

Supplementary Table 3 online only. Associations of lifestyle factors with mental health and well-being (n = 28138)

**Items**	**Mental health**										**Well-being**	
	**Depression**		**Anxiety**		**Loneliness**		**Perceived pressure**		**Self-rated health status**			
	β **(95%CI)**	***P***	***β*** **(95%CI)**	***P***	***β*** **(95%CI)**	***P***	***β*** **(95%CI)**	***P***	***β*** **(95%CI)**	***P***	***β*** **(95%CI)**	***P***
Smoking status												
Higher-risk	1 (ref)		1 (ref)		1 (ref)		1 (ref)		1 (ref)		1 (ref)	
Lower-risk	-1.19 (-1.39, -0.98)	<0.001	-0.85 (-1.02, -0.68)	<0.001	-0.18 (-0.24, -0.12)	<0.001	-0.17 (-0.28, -0.05)	0.004	2.16 (1.34, 2.97)	<0.001	1.31 (1.09, 1.53)	<0.001
Drinking status												
Higher-	1 (ref)		1 (ref)		1 (ref)		1 (ref)		1 (ref)		1 (ref)	

risk												
Lower-risk	-1.01 (-1.17, -0.85)	<0.001	-0.72 (-0.86, -0.59)	<0.001	-0.22 (-0.27, -0.18)	<0.001	-0.39 (-0.48, -0.29)	<0.001	0.47 (-0.18, 1.12)	0.158	0.52 (0.34, 0.69)	<0.001
Diet behaviors												
Higher-risk	1 (ref)		1 (ref)		1 (ref)		1 (ref)		1 (ref)		1 (ref)	
Lower-risk	-1.14 (-1.28, -1.00)	<0.001	-0.81 (-0.93, -0.69)	<0.001	-0.33 (-0.37, -0.29)	<0.001	-0.03 (-0.11, 0.05)	0.460	3.51 (2.95, 4.08)	<0.001	1.40 (1.25, 1.56)	<0.001
Physical activity												
Higher-risk	1 (ref)		1 (ref)		1 (ref)		1 (ref)		1 (ref)		1 (ref)	
Lower-risk	0.72 (0.59, 0.85)	<0.001	0.57 (0.46, 0.68)	<0.001	0.12 (0.08, 0.16)	<0.001	0.15 (0.08, 0.23)	<0.001	-1.61 (-2.13, -1.09)	<0.001	0.46 (0.31, 0.60)	<0.001
Sitting time												
Higher-risk	1 (ref)		1 (ref)		1 (ref)		1 (ref)		1 (ref)		1 (ref)	
Lower-risk	-1.13 (-1.26, -1.00)	<0.001	-0.92 (-1.03, -0.81)	<0.001	-0.28 (-0.31, -0.24)	<0.001	-0.37 (-0.45, -0.30)	<0.001	1.75 (1.23, 2.27)	<0.001	0.25 (0.11, 0.40)	<0.001
Sleep duration												
Higher-risk	1 (ref)		1 (ref)		1 (ref)		1 (ref)		1 (ref)		1 (ref)	
Lower-risk	-1.24 (-1.38, -1.11)	<0.001	-1.00 (-1.11, -0.88)	<0.001	-0.34 (-0.37, -0.30)	<0.001	-0.22 (-0.29, -0.14)	<0.001	3.24 (2.70, 3.78)	<0.001	1.55 (1.41, 1.70)	<0.001
Sleep quality												
Higher-risk	1 (ref)		1 (ref)		1 (ref)		1 (ref)		1 (ref)		1 (ref)	
Lower-risk	-3.69 (-3.86, -3.53)	<0.001	-2.93 (-3.07, -2.79)	<0.001	-0.79 (-0.84, -0.74)	<0.001	-0.73 (-0.83, -0.64)	<0.001	8.96 (8.28, 9.64)	<0.001	3.15 (2.97, 3.33)	<0.001

Note: All models were adjusted for age, sex, education level, career status, marital status, urban–rural distribution, whether having diagnosed chronic disease, family per capita monthly income, and family social status.

β, regression coefficients; *CI*, confidence interval; *ref*, reference.

Supplementary Table 4 online only. Associations of detailed lifestyle behaviors with mental health and well-being (n = 28138)

Items	Mental health										Well-being	
	Depression		Anxiety		Loneliness		Perceived pressure		Self-rated health status			
	β (95%CI)	*P*	*β* (95%CI)	*P*	*β* (95%CI)	*P*	*β* (95%CI)	*P*	*β* (95%CI)	*P*	*β* (95%CI)	*P*
Smoking status												
Current	1 (ref)		1 (ref)		1 (ref)		1 (ref)		1 (ref)		1 (ref)	
Former	0.59 (0.19, 0.98)	0.004	0.48 (0.15, 0.82)	0.005	0.24 (0.12, 0.35)	<0.001	0.41 (0.18, 0.63)	<0.001	0.09 (-1.52, 1.70)	0.913	0.99 (0.56, 1.43)	<0.001
Never	-1.33 (-1.54, -1.13)	<0.001	-0.96 (-1.14, -0.79)	<0.001	-0.21 (-0.27, -0.15)	<0.001	-0.22 (-0.33, -0.10)	<0.001	2.33 (1.51, 3.15)	<0.001	1.34 (1.11, 1.56)	<0.001
Drinking status												
Current	1 (ref)		1 (ref)		1 (ref)		1 (ref)		1 (ref)		1 (ref)	
Former	0.06 (-0.18, 0.31)	0.616	-0.01 (-0.22, 0.20)	0.929	0.07 (0, 0.15)	0.042	-0.21 (-0.35, -0.07)	0.003	-0.21 (-1.21, 0.78)	0.677	-0.25 (-0.52, 0.02)	0.066
Never	-1.21 (-1.38, -1.05)	<0.001	-0.86 (-1.00, -0.72)	<0.001	-0.28 (-0.33, -0.23)	<0.001	-0.42 (-0.51, -0.33)	<0.001	0.60 (-0.07, 1.26)	0.079	0.66 (0.48, 0.85)	<0.001
Diet scores												
0-1	1 (ref)		1 (ref)		1 (ref)		1 (ref)		1 (ref)		1 (ref)	
2	-0.48 (-0.68, -0.28)	<0.001	-0.29 (-0.46, -0.12)	0.001	-0.16 (-0.22. -0.10)	<0.001	-0.03 (-0.15, 0.08)	0.608	1.54 (0.72, 2.36)	<0.001	0.66 (0.44, 0.88)	<0.001
3	-1.15 (-1.35, -0.95)	<0.001	-0.79 (-0.97, -0.62)	<0.001	-0.36 (-0.41, -0.30)	<0.001	-0.07 (-0.18, 0.05)	0.238	3.73 (2.91, 4.55)	<0.001	1.47 (1.25, 1.69)	<0.001
4	-1.72 (-1.95, -1.50)	<0.001	-1.18 (-1.37, -0.98)	<0.001	-0.51 (-0.57, -0.44)	<0.001	-0.01 (-0.14, 0.12)	0.894	5.12 (4.20, 6.03)	<0.001	2.23 (1.99, 2.48)	<0.001
5	-2.37 (-2.67, -2.07)	<0.001	-1.65 (-1.91, -1.39)	<0.001	-0.66 (-0.74, -0.57)	<0.001	-0.07 (-0.24, 0.11)	0.447	7.41 (6.18, 8.64)	<0.001	2.73 (2.40, 3.06)	<0.001

Physical activity												
Inactive	1 (ref)		1 (ref)		1 (ref)		1 (ref)		1 (ref)		1 (ref)	
Minimally active	-0.79 (-0.99, -0.60)	<0.001	-0.62 (-0.79, -0.45)	<0.001	-0.22 (-0.27, -0.16)	<0.001	-0.08 (-0.19, 0.03)	0.137	2.91 (2.12, 3.69)	<0.001	0.88 (0.67, 1.10)	<0.001
Active	0.15 (-0.04, 0.34)	0.119	0.13 (-0.03, 0.29)	0.108	-0.03 (-0.09, 0.02)	0.247	0.09 (-0.01, 0.20)	0.084	0.46 (-0.30, 1.23)	0.236	1.09 (0.88, 1.29)	<0.001
Sitting time												
> 7 hours/day	1 (ref)		1 (ref)		1 (ref)		1 (ref)		1 (ref)		1 (ref)	
≤7 hours/day	-1.13 (-1.26, -1.00)	<0.001	-0.92 (-1.03, -0.81)	<0.001	-0.28 (-0.31, -0.24)	<0.001	-0.37 (-0.45, -0.30)	<0.001	1.75 (1.23, 2.27)	<0.001	0.25 (0.11, 0.40)	<0.001
Sleep duration												
< 5 hours/day	1 (ref)		1 (ref)		1 (ref)		1 (ref)		1 (ref)		1 (ref)	
5-6 hours/day	-1.36 (-1.65, -1.07)	<0.001	-1.00 (-1.25, -0.75)	<0.001	-0.20 (-0.29, -0.12)	<0.001	0.14 (-0.03, 0.31)	0.111	10.86 (9.67, 12.05)	<0.001	1.50 (1.17, 1.82)	<0.001
6-7 hours/day	-3.27 (-3.55, -2.99)	<0.001	-2.45 (-2.69, -2.21)	<0.001	-0.60 (-0.68, -0.51)	<0.001	-0.21 (-0.37, -0.05)	0.012	16.26 (15.13, 17.38)	<0.001	2.48 (2.17, 2.78)	<0.001
> 7 hours/day	-3.63 (-3.91, -3.35)	<0.001	-2.78 (-3.02, -2.54)	<0.001	-0.75 (-0.84, -0.67)	<0.001	-0.30 (-0.46, -0.14)	<0.001	16.37 (15.24, 17.51)	<0.001	3.51 (3.19, 3.82)	<0.001
Sleep quality												
Very bad	1 (ref)		1 (ref)		1 (ref)		1 (ref)		1 (ref)		1 (ref)	
Relatively bad	-3.56 (-3.94, -3.18)	<0.001	-2.98 (-3.31, -2.66)	<0.001	-0.64 (-0.76, -0.53)	<0.001	-1.13 (-1.35, -0.90)	<0.001	10.14 (8.57, 11.72)	<0.001	0.21 (-0.22, 0.63)	0.340
Relatively good	-6.18 (-6.53, -5.82)	<0.001	-5.03 (-5.33, -4.73)	<0.001	-1.19 (-1.29, -1.08)	<0.001	-1.57 (-1.78, -1.36)	<0.001	16.81 (15.35, 18.28)	<0.001	2.92 (2.52, 3.31)	<0.001
Very good	-7.56 (-7.92, -7.19)	<0.001	-6.13 (-6.44, -5.82)	<0.001	-1.59 (-1.70, -1.48)	<0.001	-1.84 (-2.05, -1.63)	<0.001	18.25 (16.74, 19.76)	<0.001	4.19 (3.79, 4.60)	<0.001

Note: All models were adjusted for age, sex, education level, career status, marital status, urban–rural distribution, whether having diagnosed chronic disease, family per capita monthly income, and family social status.

β, regression coefficients; *CI*, confidence interval; *ref*, reference.

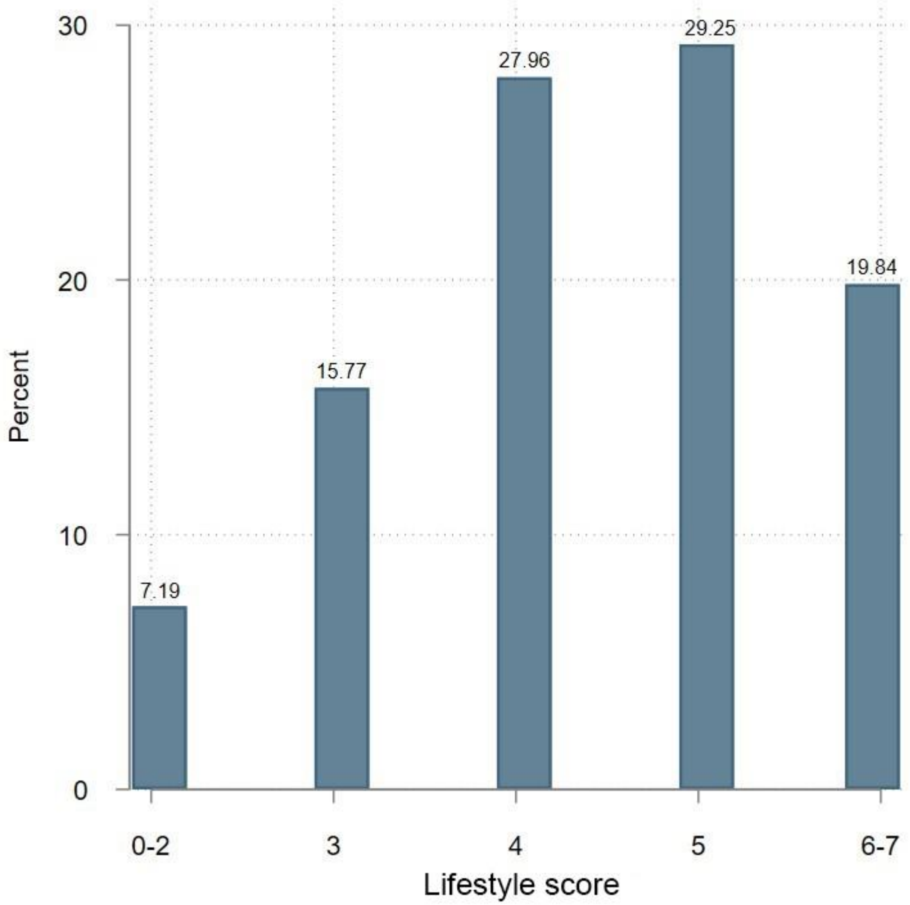

Supplementary Figure 1 online only. The population constituent ratio among different lifestyle scores.

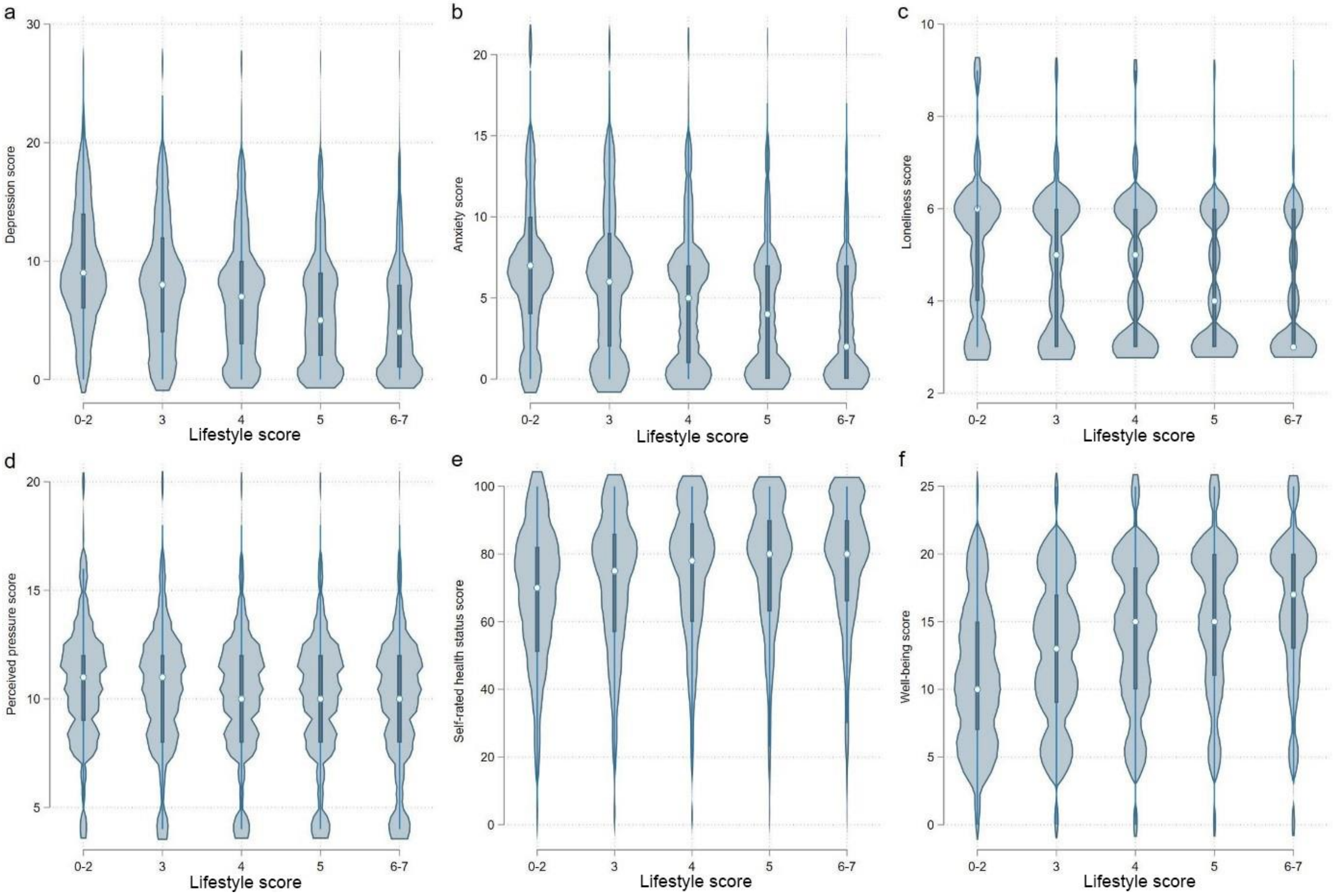

Supplementary Figure 2 online only. Distribution of mental health and well-being scores by lifestyle score.

(a) Distribution of depression score by lifestyle score; (b) Distribution of anxiety score by lifestyle score; (c) Distribution of loneliness score by lifestyle score; (d) Distribution of perceived pressure score by lifestyle score; (e) Distribution of self-rated health status score by lifestyle score; (f) Distribution of well-being score by lifestyle score.

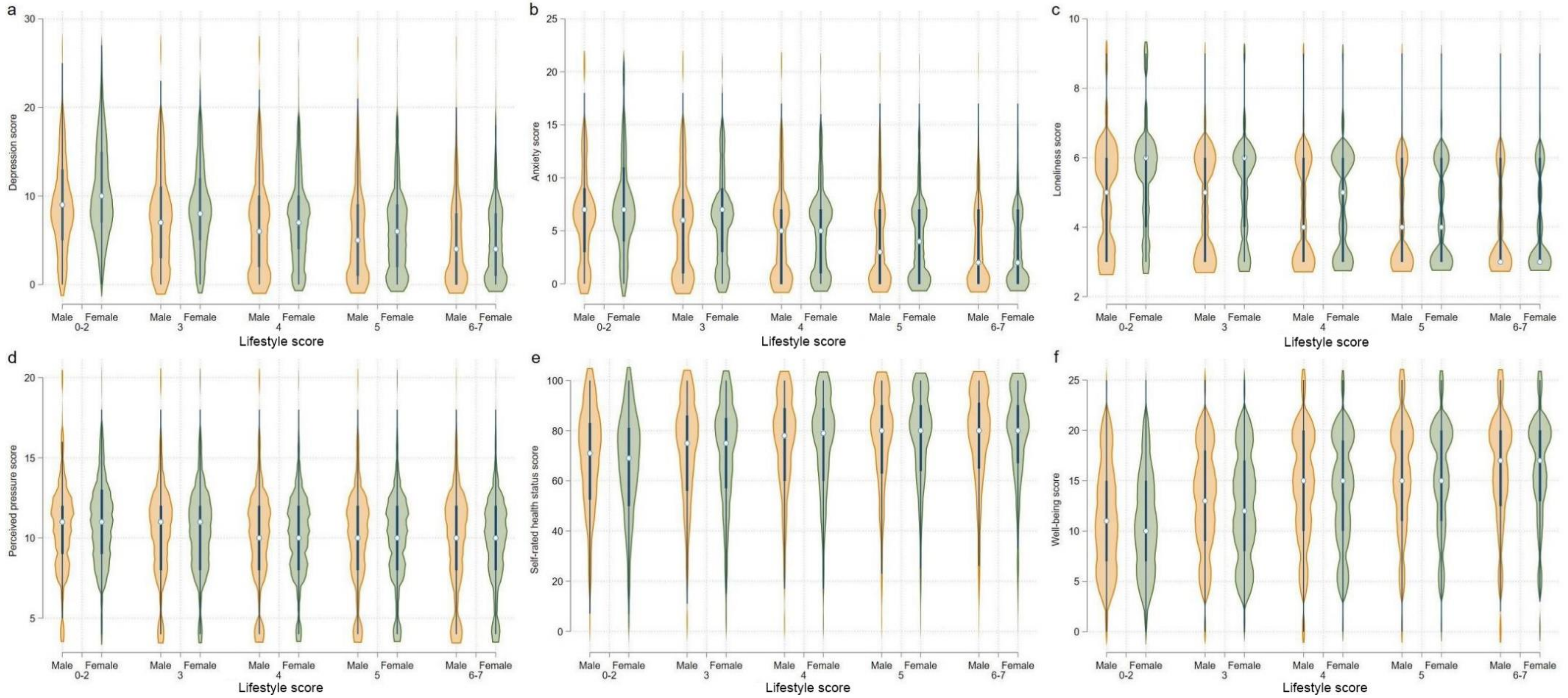

Supplementary Figure 3 online only. Distribution of mental health and well-being scores by lifestyle score stratified by sex.

(a) Distribution of depression score by lifestyle score stratified by sex; (b) Distribution of anxiety score by lifestyle score stratified by sex; (c) Distribution of loneliness score by lifestyle score stratified by sex; (d) Distribution of perceived pressure score by lifestyle score stratified by sex; (e) Distribution of self-rated health status score by lifestyle score stratified by sex; (f) Distribution of well-being score by lifestyle score stratified by sex.

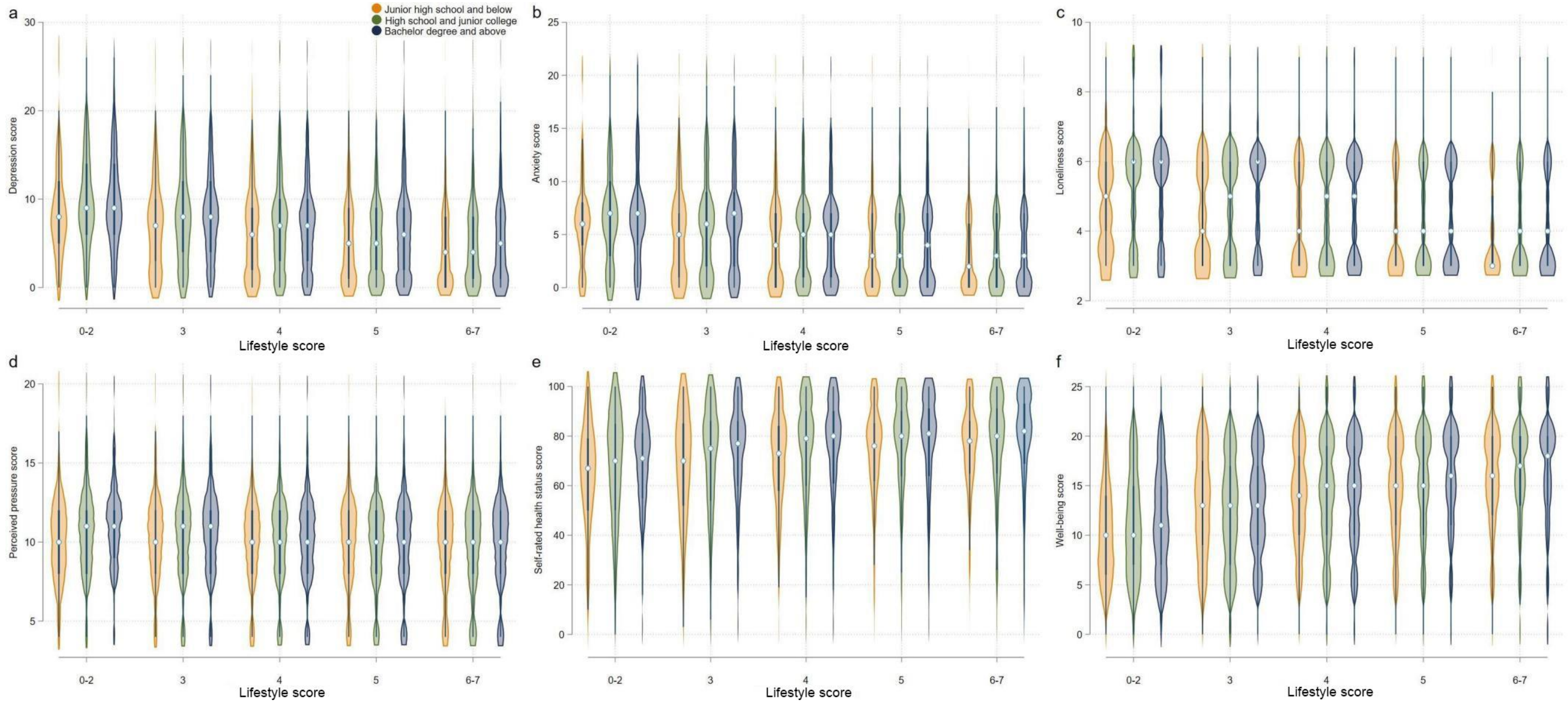

Supplementary Figure 4 online only. Distribution of mental health and well-being scores by lifestyle score stratified by education level. (a) Distribution of depression score by lifestyle score stratified by education level; (b) Distribution of anxiety score by lifestyle score stratified by education level; (c) Distribution of loneliness score by lifestyle score stratified by education level; (d) Distribution of perceived pressure score by lifestyle score stratified by education level; (e) Distribution of self-rated health status score by lifestyle score stratified by education level; (f) Distribution of well-being score by lifestyle score stratified by education level.

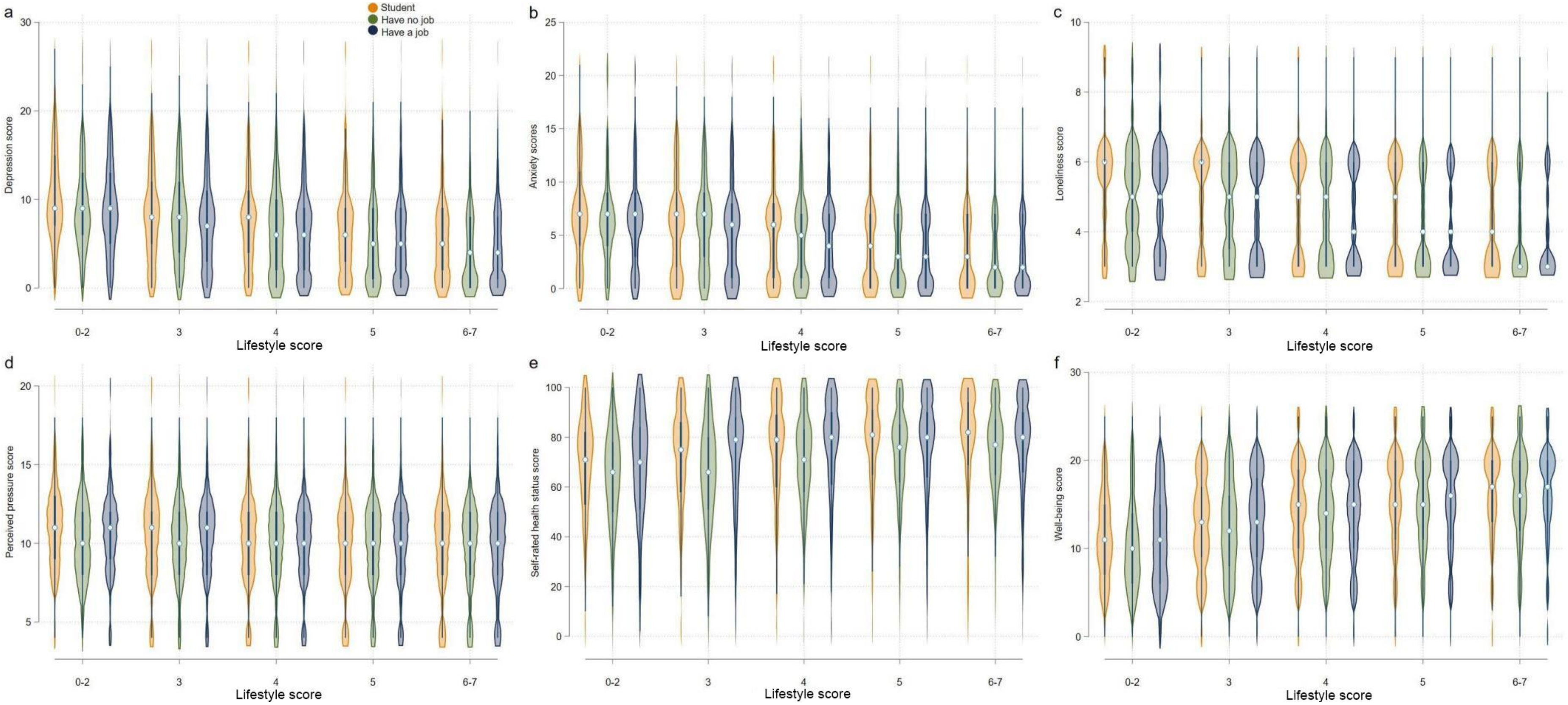

Supplementary Figure 5 online only. Distribution of mental health and well-being scores by lifestyle score stratified by career status. (a) Distribution of depression score by lifestyle score stratified by career status; (b) Distribution of anxiety score by lifestyle score stratified by career status; (c) Distribution of loneliness score by lifestyle score stratified by career status; (d) Distribution of perceived pressure score by lifestyle score stratified by career status; (e) Distribution of self-rated health status score by lifestyle score stratified by career status; (f) Distribution of well-being score by lifestyle score stratified by career status.

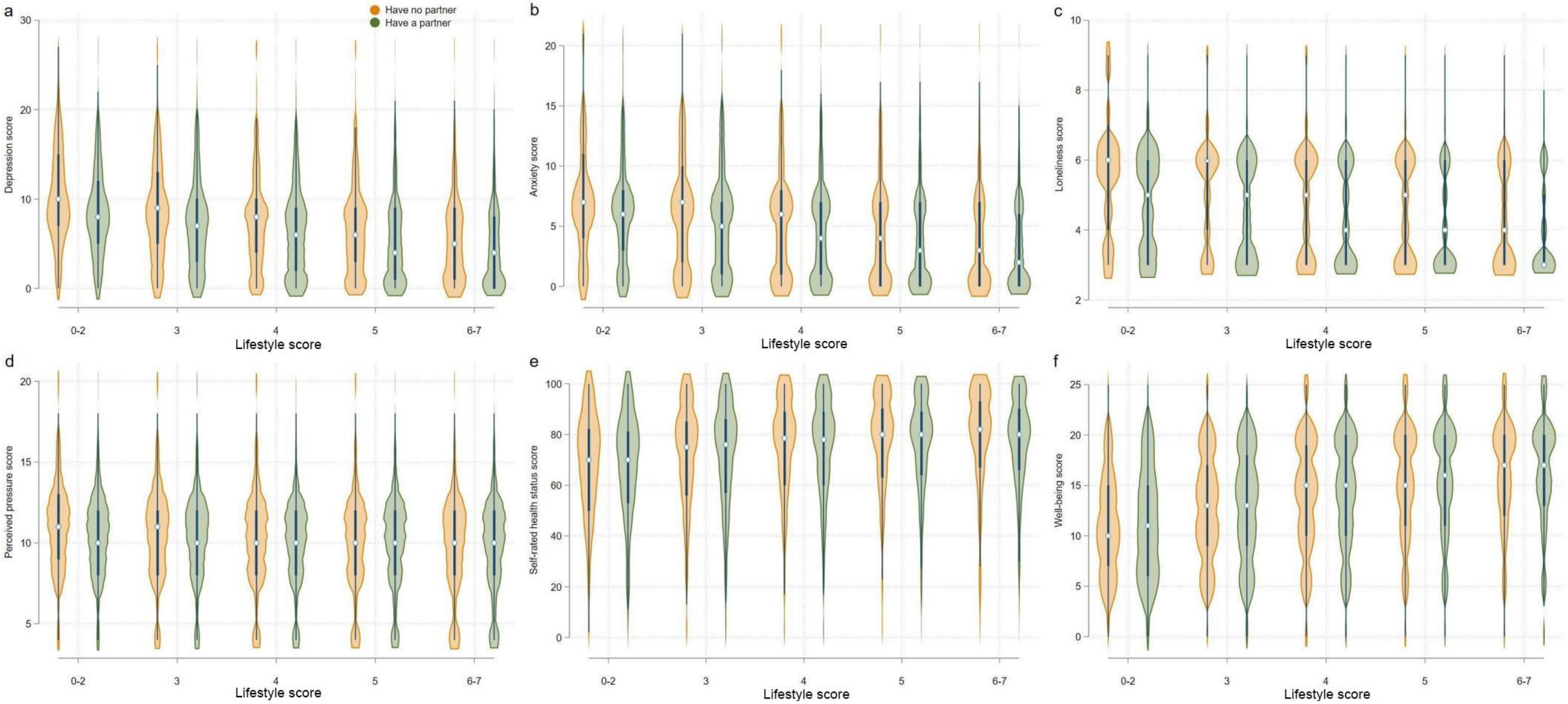

Supplementary Figure 6 online only. Distribution of mental health and well-being scores by lifestyle score stratified by marital status. (a) Distribution of depression score by lifestyle score stratified by marital status; (b) Distribution of anxiety score by lifestyle score stratified by marital status; (c) Distribution of loneliness score by lifestyle score stratified by marital status; (d) Distribution of perceived pressure score by lifestyle score stratified by marital status; (e) Distribution of self-rated health status score by lifestyle score stratified by marital status; (f) Distribution of well-being score by lifestyle score stratified by marital status.

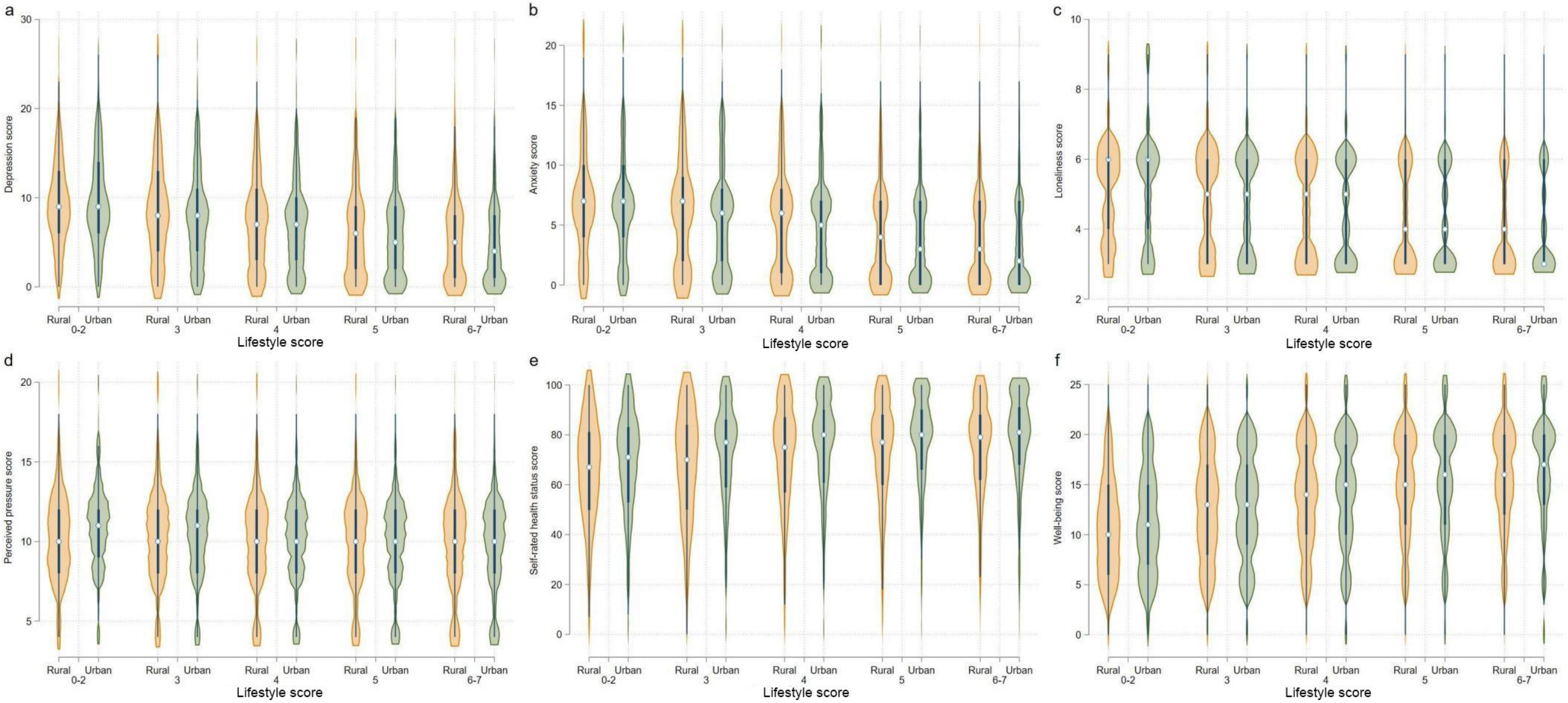

Supplementary Figure 7 online only. Distribution of mental health and well-being scores by lifestyle score stratified by urban–rural distribution.

(a) Distribution of depression score by lifestyle score stratified by urban–rural distribution; (b) Distribution of anxiety score by lifestyle score stratified by urban–rural distribution; (c) Distribution of loneliness score by lifestyle score stratified by urban–rural distribution; (d) Distribution of perceived pressure score by lifestyle score stratified by urban–rural distribution; (e) Distribution of self-rated health status score by lifestyle score stratified by urban–rural distribution; (f) Distribution of well-being score by lifestyle score stratified by urban–rural distribution.

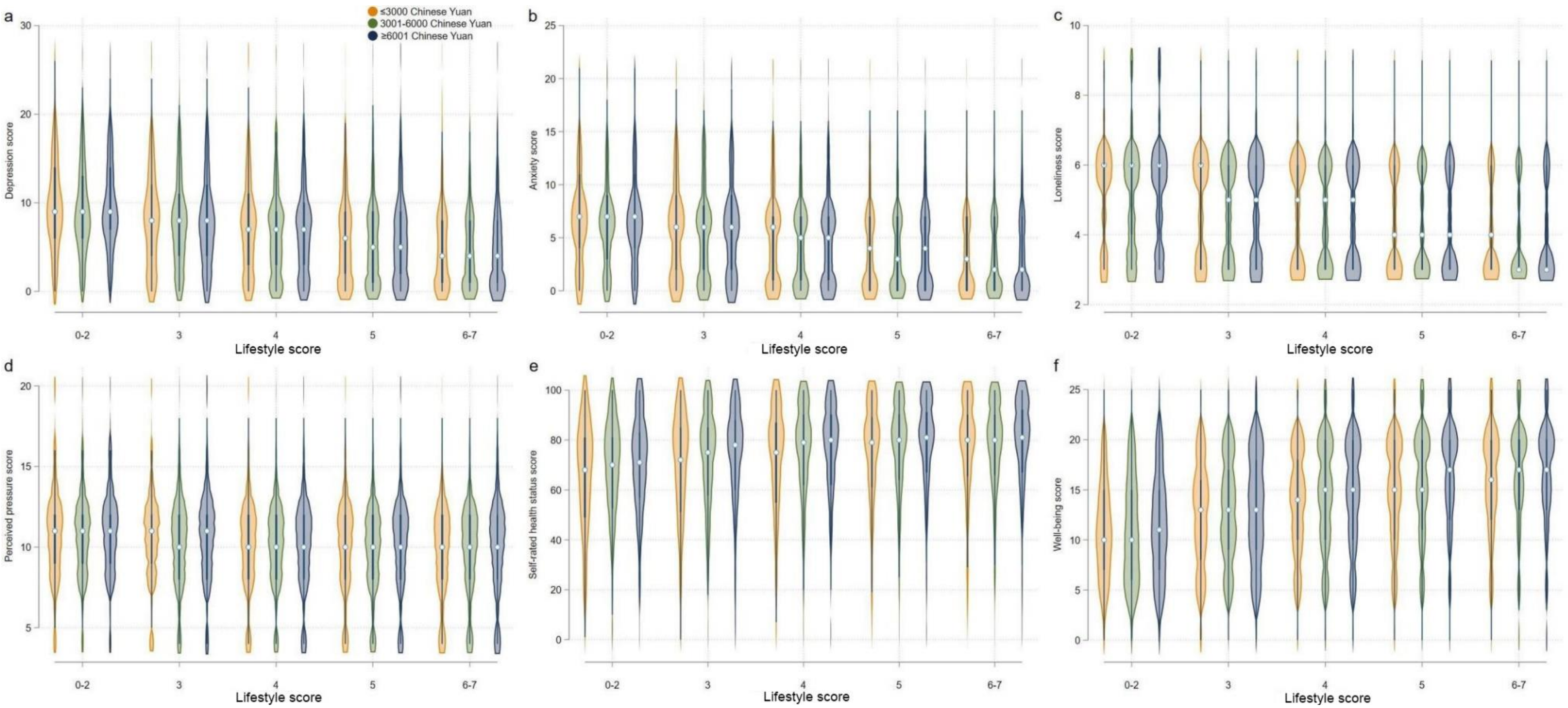

Supplementary Figure 8 online only. Distribution of mental health and well-being scores by lifestyle score stratified by family per capita monthly income.

(a) Distribution of depression score by lifestyle score stratified by family per capita monthly income; (b) Distribution of anxiety score by lifestyle score stratified by family per capita monthly income; (c) Distribution of loneliness score by lifestyle score stratified by family per capita monthly income; (d) Distribution of perceived pressure score by lifestyle score stratified by family per capita monthly income; (e) Distribution of self-rated health status score by lifestyle score stratified by family per capita monthly income; (f) Distribution of well-being score by lifestyle score stratified

by family per capita monthly income.

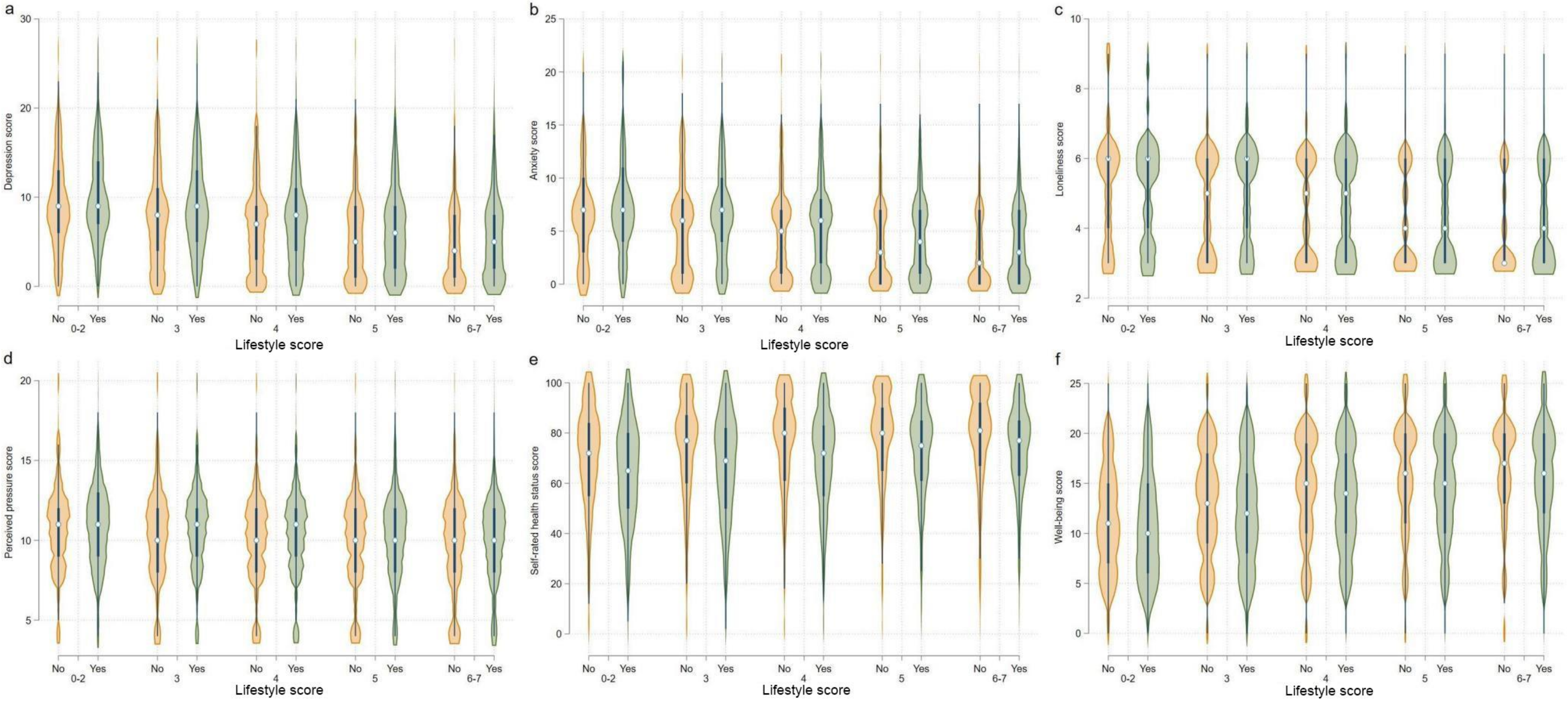

Supplementary Figure 9 online only. Distribution of mental health and well-being scores by lifestyle score stratified by whether having diagnosed chronic disease.

(a) Distribution of depression score by lifestyle score stratified by whether having diagnosed chronic disease; (b) Distribution of anxiety score by lifestyle score stratified by whether having diagnosed chronic disease; (c) Distribution of loneliness score by lifestyle score stratified by whether having diagnosed chronic disease; (d) Distribution of perceived pressure score by lifestyle score stratified by whether having diagnosed chronic

disease; (e) Distribution of self-rated health status score by lifestyle score stratified by whether having diagnosed chronic disease; (f) Distribution of well-being score by lifestyle score stratified by whether having diagnosed chronic disease.
